# Accessing Beyond-Light Line Dispersion and High-*Q* Resonances of Dense Plasmon Lattices by Bandfolding

**DOI:** 10.1021/acsphotonics.4c02323

**Published:** 2025-01-07

**Authors:** Nelson de Gaay Fortman, Debapriya Pal, Peter Schall, A. Femius Koenderink

**Affiliations:** † Institute of Physics, University of Amsterdam, 1098 XH Amsterdam, The Netherlands; ‡ Department of Physics of Information in Matter and Center for Nanophotonics, NWO-I Institute AMOLF, Science Park 104, NL1098XG Amsterdam, The Netherlands

**Keywords:** lattice plasmons, nanolaser, bound states in the continuum, hexagonal lattice, K-point

## Abstract

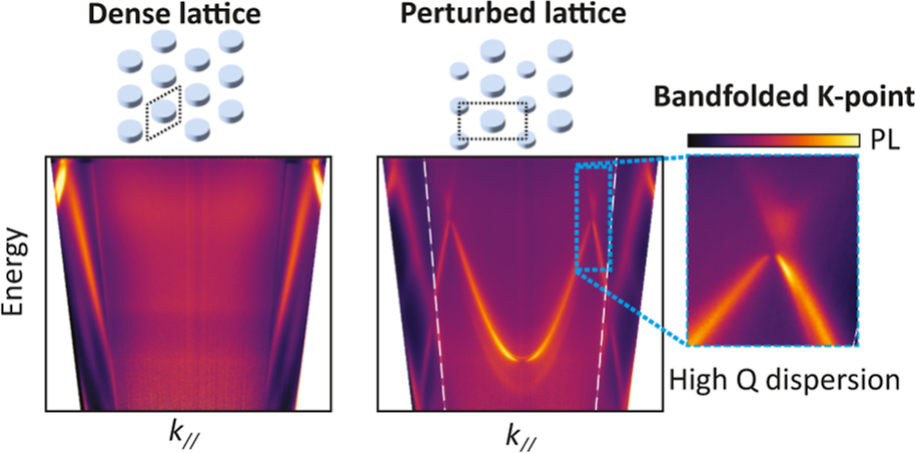

Dense plasmon lattices are promising as experimentally accessible implementations of seminal tight-binding Hamiltonians, but the plasmonic dispersion of interest lies far beyond the light line and is thereby inaccessible in far-field optical experiments. In this work, we make the guided mode dispersion of dense hexagonal plasmon antenna lattices visible by bandfolding induced by perturbative scatterer size modulations that introduce supercell periodicity. We present fluorescence enhancement experiments and reciprocity-based T-matrix simulations for a systematic variation of perturbation strength. We evidence that folding the *K*-point into the light cone gives rise to a narrow plasmon mode, achieving among the highest reported quality factors for plasmon lattice resonances in the visible wavelength range despite a doubled areal density of plasmon antennas. We finally show *K*-point lasing and spontaneous symmetry breaking between the bandfolded *K*- and *K*′-modes, signifying that intrinsic symmetry properties of the dense plasmon lattice are maintained and can be observed upon band folding.

## Introduction

Two-dimensional (2D) periodic photonic systems, such as photonic crystals, plasmonic lattices, metasurfaces, and waveguide arrays, are an interesting arena to study the effects of symmetry and symmetry breaking on wave interactions. For instance, the interaction of light with periodically arranged photonic systems can simulate a plethora of classical solid-state tight-binding Hamiltonians, where control over photonic design allows for versatile control of interaction strengths, symmetries, and defects. This freedom of design combines advantageously with the ability to perform high-resolution imaging of photonic systems in real space and *k*-space, as well as in the spectral and temporal domain. Example achievements include topological Dirac cone band structures with photonic Hall edge state effects,^1−5^ exotic topologies due to parity-time (PT) symmetric lattices,^6−8^ and, more recently, photonic Landau levels in strained crystals.^9,10^ In another strand of nanophotonics research, the focus is on the interplay of symmetries and resonant scattering. For instance, Mie-resonant meta-atoms, as well as plasmonic nanoparticles, give access to Fano-resonances and bound states in the continuum (BIC) physics.^11^ These resonances have implications for strong light–matter interaction, nonlinear optics, and nanolasers. For instance 2D lattices with gain are studied in the context of plasmon lattice lasers,^12−18^ PT-symmetry,^6,8,19^ BIC-based lasers, and topological lasing.^7,20,21^

In the context of 2D periodic lattices of resonant scatterers, there is particular interest in lattices with short interparticle distances with respect to free space radiation wavelength, i.e., subwavelength pitch. For instance, many theoretical reports^5,7,8,22−25^ have addressed plasmon particle lattices with strong 1/*r*^3^ near-field interactions as possible classical analogs of tight-binding Hamiltonians. Short distances provide strong nearest-neighbor interactions and, at the same time, forbid diffractive coupling to the radiation continuum. This should be contrasted to the limit of large periodicities. In this limit, the physics is not dominated by nearest-neighbor interactions but by surface lattice resonances (SLR)—collective oscillations in which localized resonances (either plasmonic or Mie resonances) hybridize with grating anomalies. According to theory, dense plasmonic lattices imbued with gain can be designer realizations of non-Hermitian Hamiltonians with physics typical for the field of PT (parity-time) symmetry breaking and topology.^7,8,19^ For instance, in kagome plasmon lattices, plasmonic interactions are expected to express in optical angular momentum-coupled chiral topological edge modes.^26^ In active honeycomb lattices, symmetry-broken unit cells are predicted to lead to topological chiral lasing.^7^ At PT-symmetry breaking conditions in such lattices, *K*-point Dirac cones are expected to evolve into rings of exceptional points,^8^ where the mode structure becomes pseudochiral. Despite these interesting theory proposals, experimentally dense plasmonic lattices have hardly been addressed. Experiments on plasmon lattices have largely focused on the opposite regime, i.e. large periodicities, diffractive resonances, and the benefits of surface lattice resonances (SLRs) for sensing, light emission, and lasing.^12,14,15,17,18^ In such conventional plasmon lattice scenarios, the band structure of interest can be directly mapped by far-field Fourier microscopy in a high numerical aperture (NA) microscope. A key practical obstacle toward studying dense, strongly interacting lattices is that the proposed physics pertains to deeply subwavelength pitches, resulting in Brillouin zones that extend well beyond the light line. Since the lattice modes of interest have wave vectors that lie well beyond the light line they cannot be accessed using standard far-field microscopy.

In this paper, we explore beyond-the-light-line band structures of dense plasmonic lattices through a technique called band folding.^8,19^ The core idea is the following: starting from a dense lattice of subwavelength pitch, one applies a periodic supercell of slight perturbations in particle size. The reciprocal lattice vectors of the supercell then fold the original beyond-the-light line dispersions back into the light cone. Band folding has been applied to surface plasmon polaritons^27−29^ and photonic crystal slabs,^30−39^ and is currently receiving attention in the community of dielectric metasurfaces as a route to creating quasi-BICs by symmetry breaking.^11,40−42^ In this work we perform a systematic simulation and experimental study of the effect of band folding on the full band structure of dense plasmon metasurfaces, studying both fluorescence and gain scenarios. While there are studies of plasmonic lattices with bipartite unit cells^43−53^ and superlattice perturbations^54,55^ only ref (56) investigated beyond-the-light-line physics of a dense plasmon lattice. We demonstrate that it is indeed possible to fold beyond-the-light-line dispersions into the observable light cone and identify the role of the strength of the supercell perturbation. Our experiments show that in certain folding scenarios, cancellation mechanisms occur in the radiative damping of modes that are reminiscent of reported mechanisms for the formation of BICs in dielectric metasurfaces^11^ and that give rise to surprisingly high *Q*-factor resonances. For instance, we find that perturbed dense lattices can exhibit *Q*-factors that are 4 to 5 times higher than dilute reference lattices with the same supercell despite having twice the areal density of lossy metal particles. At these conditions, we show plasmon lattice lasing. Folding strategies that leave the original degeneracy of symmetry points intact furthermore are shown to preserve the property of spontaneous symmetry breaking in the lasing behavior, showing that it is possible to preserve main characteristics of the mode structure of the dense lattice despite the folding perturbation. Our work offers a practical route for studying plasmonic counterparts of topological and PT-symmetric Hamiltonians.

## Bandfolding Strategies

**Figure 1:**
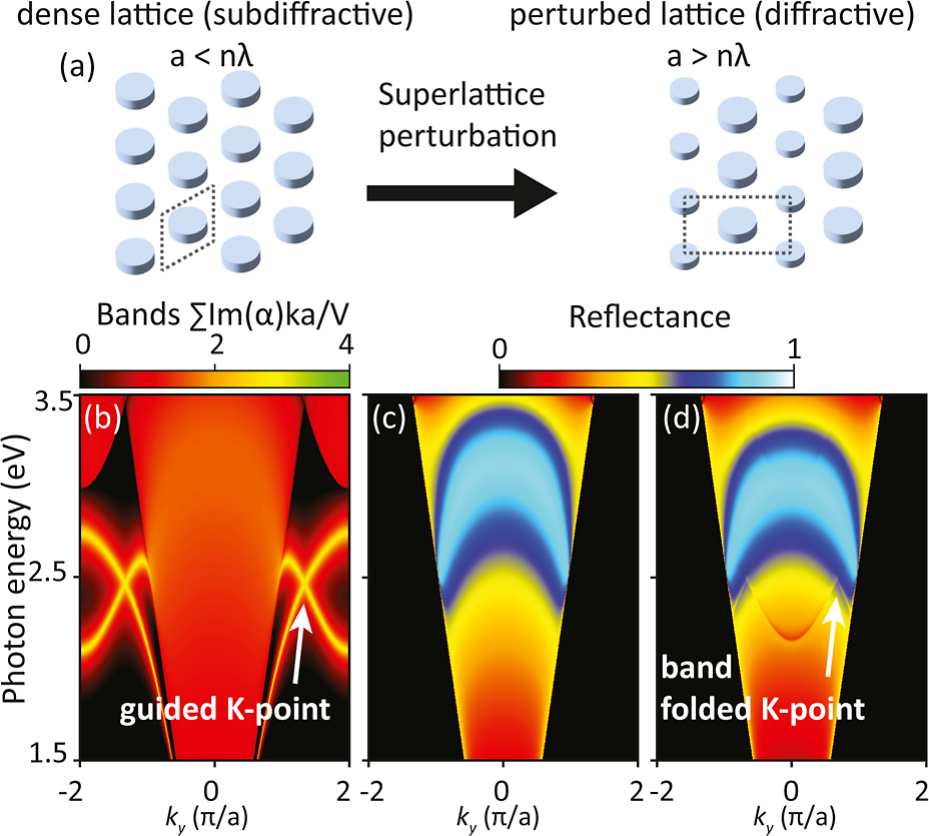
Bandfolding by supercell perturbation. (a) The dense lattice has subdiffractive pitch, so we perturb the lattice by antenna size modulations that introduce a supercell with diffractive period. (b) Band structure for a dense plasmon lattice (*a* = 160 nm) as visualized by the sum of the imaginary part of the effective eigen polarizabilities dressed with lattice interactions in a simple point dipole model—a metric that generalizes the notion of extinction. To bring out only the in-plane band structure we assume oblate plasmonic ellipsoids (diameter in *xy*-plane 112 nm, volume *V* = 3 × 10^–22^ m^3^). The *K*-points are in the guided regime well beyond the light line, not accessible from the far field. (c) Reflectance of the dense lattice shows no photonic bands within the far field radiation cone except for the broad, superradiantly damped collective plasmon mode at the energy of around 3 eV. (d) Bandfolding induced by perturbing particles of the supercell (12% decrease in *x*–*y* diameter). Bloch modes around the *K*-point become visible due to bandfolding.

The main idea of band folding is conceptually visualized in Figure 1: the sublattice is periodically perturbed such that diffractive interactions become observable in the far field. As starting point (panel (a)), we consider a subdiffractive hexagonal lattice, meaning that the lattice constant *a* = 160 nm is not large enough for diffractive coupling of far-field radiation to in-plane surface lattice resonances. The lattice vectors of the dense hexagonal lattice are  and *a*_2_ = (0, *a*), and the reciprocal lattice vectors:  and . To illustrate the basic concept of folded band structures and observables in such a system, we evaluate the minimal coupled-dipole model^57,58^ to calculate “extinction” band structures in Figure 1b,c,d. The model (see Methods for details) ignores both multipole scattering and stratified dielectric surrounding. In the calculated band structure of the dense lattice, Figure 1b, we observe a strongly damped band that is blue-shifted compared to the single particle resonance at the Γ-point, redshifting toward the light-cone, as the lattice is subject to radiative damping by plasmon hybridization.^59^ Beyond the light cone, sharp bands arise. Here, the collective modes have no radiation damping, and the loss of the resulting guided lattice modes is determined by the mode overlap of the field with the absorptive particles. Band crossings occur at the first order *K*-points, i.e., beyond the observable light line. Figure 1c reports a concomitant optical observable, namely far-field reflectivity. The area beyond the light cone is not accessible, while within the light cone, the superradiantly damped dispersion is evident. Next we implement bandfolding by periodically perturbing the dense lattice. By decreasing particle diameters in every other unit cell along just the *y* direction (Figure 1a) we form a rectangular superlattice with reciprocal lattice vectors that should fold the beyond-the-light-line dispersion back inside the light line, Figure 1d. Indeed, a replica of the bands near the guided *K*-point that were originally only visible beyond the light line (panel (c)) now become clearly visible in the calculated reflectivity. The perturbation strength controls the contrast of this feature. This simple calculation highlights that bandfolding gives access to beyond-light line dispersion features. At the same time, the model points at a complication to anticipate in folding experiments: the bands in Figure 1d have asymmetric, Fano-type lineshapes due to mixing of the folded features with direct reflectivity signal.

**Figure 2:**
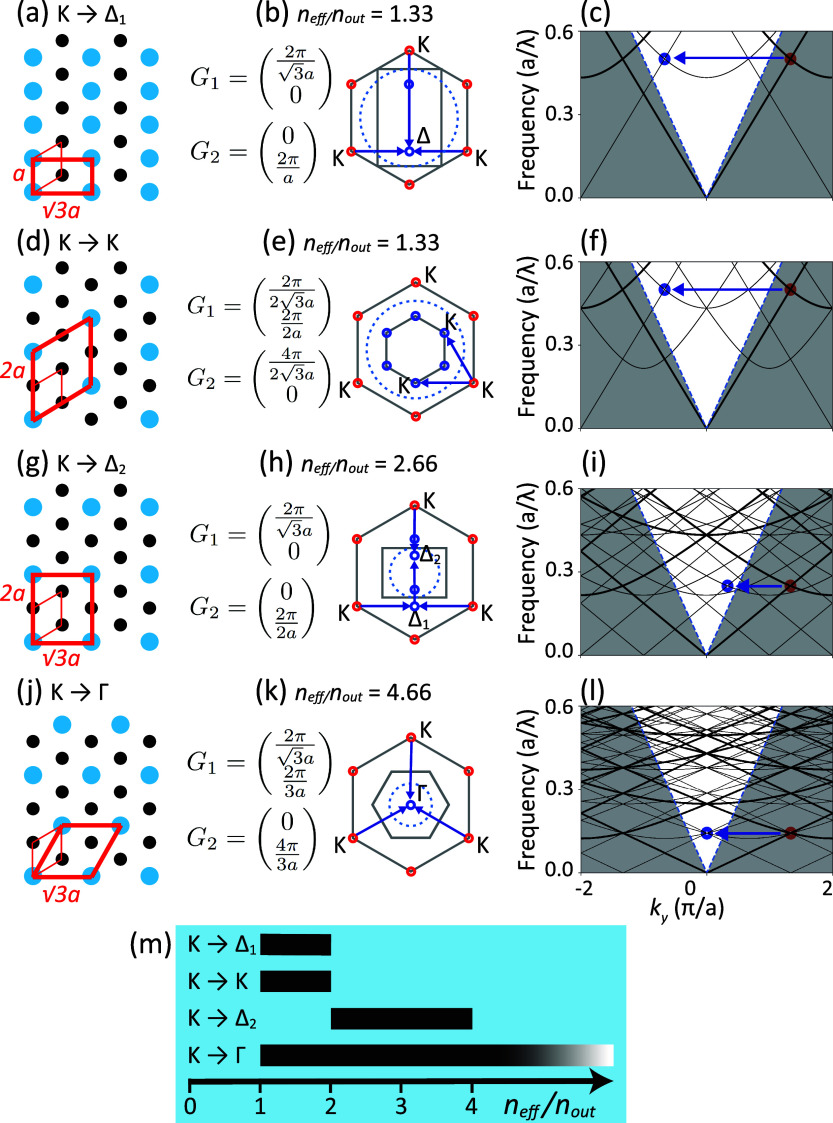
Different bandfolding strategies, all starting from a hexagonal dense lattice of lattice constant *a*. Panel (a): rectangular superlattice ( long in the *x*-direction) reducing the symmetry from *C*_6_ to *C*_2_. (b) Reciprocal lattice vectors of the rectangular superlattice repeat three guided *K*-points (corners of the first Brillouin zone indicated with red circles) at the Δ-point (blue circle). Blue dotted circle: slab light cone. (c) Folded free-photon dispersion for an effective mode index *n*_eff_/*n*_out_ = 1.33. Bands of the dense lattice (thick black lines) repeat upon superlattice perturbation (thin black lines). (d–f) Folding strategy where *C*_6_ symmetry is preserved and 6 *K*-points emerge inside the light line. (g–i) Rectangular superlattice with large lattice constants, required when dealing with larger mode indices. (j–l) Strategy that folds *K* and *K*′ onto each other and onto the Γ-point. (m) Overview of which folding strategies to choose depending on the effective mode index *n*_eff_ relative to the outside medium *n*_out_.

Aside from choosing the perturbation strength, a nontrivial choice is that of folding periodicity. Two main considerations are pertinent. The first question is how far beyond the light line one wishes to access the dispersion relation. In essence, this consideration requires matching the mode index of the band to be folded with the supercell periodicity, i.e., with the reciprocal lattice vector offered by the perturbation. The second consideration is the symmetry reduction of the superlattice compared to the underlying structure that one tolerates. For instance, in the example of Figure 1, a rectangular supercell is used, which evidently reduces the symmetry relative to that of the hexagonal lattice. An excellent and general overview of symmetry considerations for folding has been reported by Overvig et al.^11^ on the basis of group representation theory and in the context of designing bound states in the continuum modes. Here, we limit ourselves to discussing practical constraints on supercell periodicity. In Figure 2, we display four elementary examples of band folding symmetries that bring a guided *K*-point mode into the light line, taking the case of hexagonal lattices as an illustrative example. In typical plasmonic and dielectric metasurface scenarios, one could envision that the dispersion of the dense lattice (no superlattice perturbation) is to first order like that of a waveguide of mode index *n*_eff_, which is repeated at every reciprocal lattice vector. This description is common in the description of, e.g., plasmon lattices in waveguides,^58^ and also applies to, e.g., perforated dielectric membranes.^60^ While plasmon hybridization or photonic crystal effects will generally shift bands, we use this nearly free-photon repeated zone scheme dispersion to illustrate the considerations for folding strategies.

A first folding strategy (panel a) is to introduce a rectangular supercell (lowering the lattice group symmetry of *C*_6_ to *C*_2_), with pitch along *y* equal to that of the dense lattice, but along *x* larger than that of the dense lattice by . A second strategy (panel d) maintains the *C*_6_ symmetry upon perturbation by choosing as superlattice a hexagonal lattice with twice the pitch of the original. Figure 2b,e shows the Brillouin zones of the dense lattice (hexagon spanned by red circles) and the superlattice (smaller gray polygon). The dotted line is the light cone set by the index of the surrounding medium. For the rectangular strategy, the three beyond-the-light-line *K*-points (top one at *k* = (0, 4π/3*a*) μm^–1^) are repeated by reciprocal lattice vectors of the supercell at a single Δ-point (*k* = (0, – 2π/3*a*) μm^–1^) inside the light line, while the *K*′-points fold toward Δ′. This is in contrast to the second strategy, where after folding there are three *K*-points and three *K*′-points inside the light line (Figure 2e). In both superlattice symmetries, the original property that the diffractively coupled *K*-points are uncoupled from the *K*′-points remains intact upon folding. Band diagrams in Figure 2c,f show the dispersion of the dense lattice—visible as the thicker set of black lines—repeated by the superlattice periodicity (replicas indicated as thinner lines).

An important observation from Figure 2 is that a given folding strategy only works for a particular range of mode indices. Raising the mode index essentially rescales the frequency axis, moving down the band crossings while maintaining them at the same *k*-space coordinate. Evidently, at some point this will drop the replica (blue circle in panels (c,f)) below the light cone of the surrounding medium. For the scenarios at hand this occurs at a mode index contrast above 2. Instead, one needs a smaller superlattice reciprocal lattice vector or, equivalently, a larger supercell. This is demonstrated in the third row of Figure 2: the strategy with a rectangular supercell that is effective up to a 4× larger mode index than that of the surrounding medium. This folding strategy retains the property that the triplet of *K*-points, and the triplet of *K*′-points each fold to distinct special points of the rectangular superlattice (Δ_1,2_-points). Figure 2m summarizes these considerations: any mode index at hand needs to be matched to a specific supercell choice to ensure that one actually folds into the light cone. The one exception is if the superlattice folds the *K*-points toward the Γ-point, which by construction is always in the light cone. This occurs when the superlattice is hexagonal but rotated by 90° and with  larger pitch than the dense lattice. With this folding the *K* and *K*′ become coupled. This folding design is common in the field of topological photonic crystals.^3,10^

## Measurement of Realistic Dense Plasmonic Lattices

We perform fluorescence-based, spectrally resolved Fourier microscopy experiments on dense metasurfaces with folding perturbations. Using electron beam lithography and a liftoff process, we fabricated plasmonic metasurface arrays of constituent cylindrical shaped particles, each 35 nm high but with varying diameters (see Supporting Information with fabrication procedure and SEM images). The silver particle arrays on glass are embedded in a 450 nm dye-doped polymer waveguide layer via spin coating with SU8, which is doped with 0.5 wt % rhodamine 6G. As a basis, we take the typical sample structure employed in studies of plasmon-enhanced fluorescence and lasing^13,61,62^ but designed with subwavelength pitch. This standard geometry has particle lattices fabricated on glass, embedded in a 2D polymer layer that functions both as a waveguide and as a host for the active medium. The waveguide thickness is generally chosen such that in the absence of particles it behaves as a 2D waveguide with a single transverse electric (TE) and a single transverse magnetic (TM) mode. These guided modes dominate the waveguide’s local density of states (LDOS), which is beneficial for capturing fluorescence and facilitating lattice resonance formation. As we deal with a mode index of 1.55 on top of glass (*n* = 1.45), our effective mode index contrast is *n*_eff_/*n*_out_ = 1.07. Therefore, we choose the first folding strategy in Figure 2a,b,c. Using an inverted microscope, we excite the dye polymer layer with a 250 fs pulsed pump laser at 515 nm wavelength, with a 1 MHz repetition rate. The back focal plane of the objective is projected onto an imaging spectrometer slit to map the dispersion diagram. The dispersion diagrams are shown as photoluminescence enhancement (PLE), obtained by normalizing the data to similar dispersion diagrams measured on sample area without a metasurface. Experimental details can be found in Methods, and the fabrication setup in the Supporting Information.

**Figure 3:**
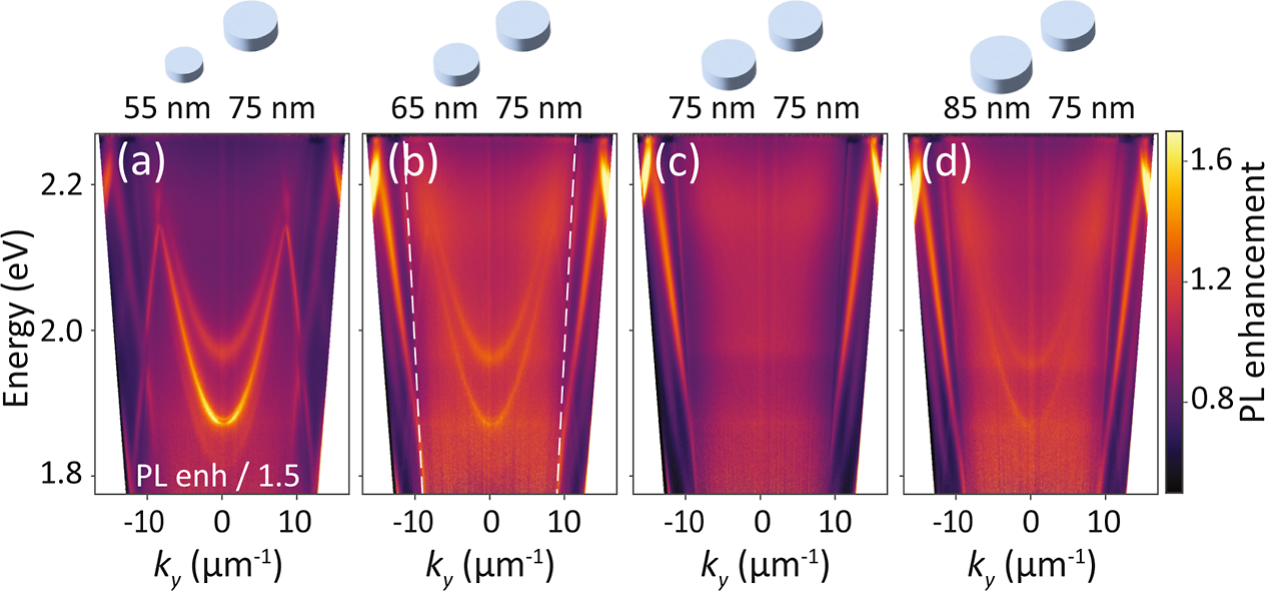
Measured fluorescence enhancement band structures of the unperturbed hexagonal dense lattice (c), and rectangular supercell diameter perturbations in (a), (b) and (d).

Three sample geometries are relevant in our experiment. These are first, the unperturbed subdiffractive hexagonal lattice, referred to as the “dense lattice” from here on, second, the rectangular diffractive reference lattice, referred to as “empty superlattice”, and last the perturbed lattice that should show band folding, which we will refer to as “dense perturbed lattice”. In Figure 3a–d, we observe the progression of fluorescence band structures as we systematically perturb the superlattice particle’s diameter from 55 to 85 nm in 10 nm increments while keeping the other particle’s diameter fixed at 75 nm. We find that at zero perturbation, the dense lattice case, no bands are visible except for quasi-guided modes at an effective mode index of around 1.2. These quasi-guided modes are not particularly related to the plasmon antenna resonances, as they already occur in a simple multilayer model for a polymer waveguide on a thin reflector. Upon introducing superlattice perturbation, folded bands emerge in the dense perturbed lattices, and higher perturbation strength corresponds to increased contrast. In the Supporting Information, all salient band structure features are reproduced and explained by full-wave simulations with the open-source software code *treams* based on the T-matrix method by Beutel, Fernandez-Corbaton and Rockstuhl,^63^ where we employ reciprocity^64−67^ to relate plane wave far-field angular emission to absorption calculations of incident waves. Note that we do not mean physical absorption by the dye molecules, but instead numerical absorption in the polymer with a small value of nondispersive κ = 0.006. As ref (64) discusses, this absorption is used as the time inverse of emission in reciprocity-based calculations. From the simulations we learn that the parabolic bands derive from interaction of the particles with the TM waveguide mode (parabola in *k*-space with minimum at 2.0 eV, no observable stop band) and bandgapped TE bands (lower energy compared to TM band). The TE modes have the brightest intensity in measured band structures, as the waveguide field aligns with the mainly in-plane nanoparticle polarizability tensor. Polarization-resolved measurements with a linear polarizer crossed to the spectrometer slit confirm the TE/TM mode assignment from theory (data shown in Supporting Information). On a final note, in the Supporting Information, we report band structure measurements for band folding through the second strategy displayed in Figure 2 (row 2, *K* folds to *K*). Importantly, we find that dispersions near the *K*-points are very similar to the bandfolded dispersions near the Δ-points, observed in Figure 3. This means that different folding strategies may be employed to image the same truly guided mode, and that deformation of the original guided dispersion is limited.

**Figure 4:**
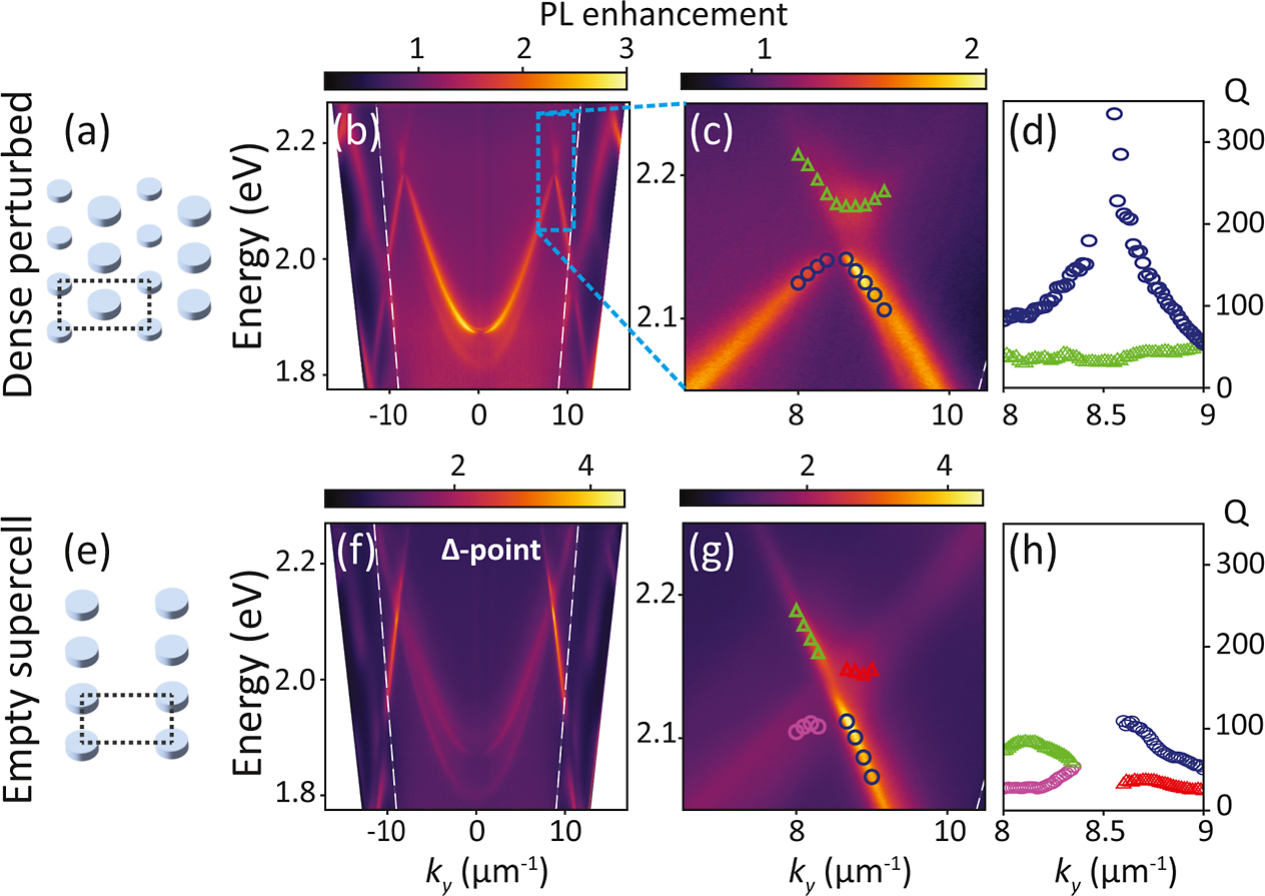
Quality factor analysis for bands near the Δ-point/folded *K*-point. (a) The dense perturbed lattice geometry and (b) its measured fluorescence enhancement band structure, where a linear polarizer with horizontal orientation was placed in front of the spectrometer slit. (c) Zoomed image of the crossing of Bloch modes with the dark spot at the Δ-point. (d) Measured quality factors were obtained by fitting Lorentzian lineshapes through vertical crosscuts in panel (c), with a maximal value of *Q* = 330. The green triangles and blue circles highlight *Q*-factors along different dispersion branches. (e–h) Results for the rectangular empty superlattice. The dense perturbed lattice shows up to 3 times higher *Q*, near Δ-points than empty superlattice, despite twice as much metal in the system.

A most remarkable feature of the folded band structure arises near the folded *K*-points, and becomes apparent when analyzing the spectral width of the folded bands. Figure 4 shows the dispersion diagrams for the dense perturbed lattice (with the superlattice particle diameter of 55 nm reproduced from Figure 3a) alongside that of the empty rectangular superlattice (panel b versus f). Close-ups near the Δ-points/folded *K*-points reveal a marked difference: in the dense perturbed lattice, there is a clear gap around 2.15 eV at *k*_*y*_ = 8.5 μm^–1^. Additionally, the lower band appears dark right at the special point. This contrasts with the empty superlattice (panel g): at the Δ-point bands cross, but no avoided crossing or dark area is observed. In a purely free photon picture, i.e., at zero scattering strength, the location of the rectangular lattice bands and the folded dense hexagonal lattice bands would simply overlap at the Δ-point. The difference thus points at the much stronger particle interactions in the dense lattice, which become visible upon bandfolding. To quantify the sharpness of the bands, we evaluate the quality (*Q*) factors by fitting double-Lorentzian curves to vertical crosscuts through the dispersion data (see Supporting Information for the fitting procedures), where even the smallest recorded widths of 1.7 nm are still a factor 4 above our spectral resolution. In Figure 4d,h, we plot the extracted *Q*-factors as a function of in-plane momentum *k*_*y*_ for the dense perturbed lattice and empty superlattice, with blue circular and green triangular markers representing the lower and higher energy bands. The fitted center frequencies extracted from the fitting analysis are overlaid on the zoomed ω–*k* plots in Figure 4c,g, displaying only every eighth marker for visual clarity. Remarkably, the *Q*-factor reaches values as high as *Q* = 330 near the Δ-point. In comparison, the empty superlattice does not exhibit a *Q*-increase near the special point, with a maximum *Q*-factor of only around 110 that is typical of standard diffractive plasmon lattices.^18^ This observation is both counterintuitive and surprising: despite containing twice the amount of metal per unit cell, the dense lattice displays a *Q*-factor up to three times that of the empty superlattice. Additionally, a *Q*-factor of 330 is exceptionally high. While reported record *Q*-factors^68^ for plasmon lattice resonances are on the order of 10^3^, such high *Q*-factors are only achieved at telecom wavelengths (where metal loss is minimized) and observing them usually requires coherent illumination with a very small angular spread, addressing very large metasurface areas. In contrast, our measurement stems from incoherent emission from randomly located and mutually incoherent dipole sources within a relatively small metasurface footprint of only 100 μm across and emitting at 590 nm, where the particle albedo is low. Similar to ref (69), our measured *Q*-factor of 330 near the Δ-point is among the highest measured in the visible wavelength range,^18,43,70^ and is especially remarkable at this large areal density of metal.

## Origin of the High-*Q* Dark Feature at the Folded *K*-point

**Figure 5:**
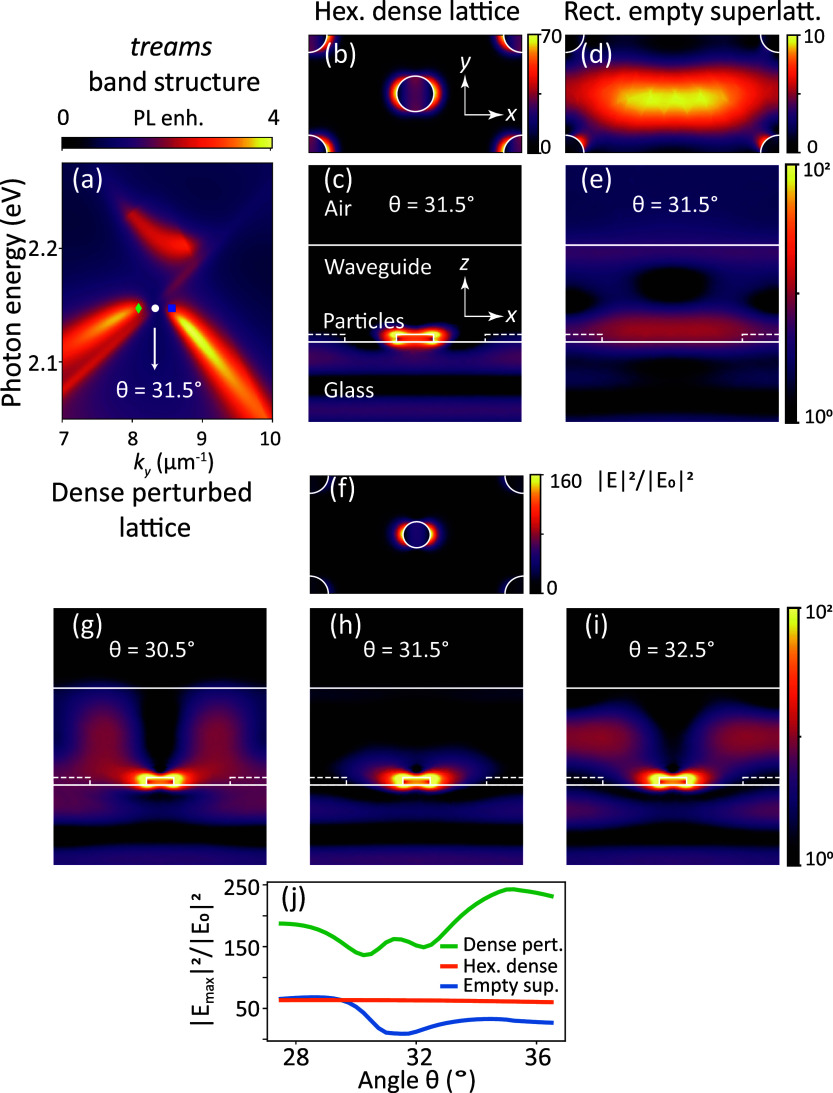
(a) T-matrix based *treams* simulation of band structure near the dark spot for the bandfolded *K*-point. Near-field intensity enhancement (|**E**|^2^/|**E**_0_|^2^) in the *xy* and *xz* planes (cuts midway through the central particle) for (b,c) a hexagonal dense lattice and (d,e) a rectangular superlattice at θ = 31.5° incidence angle at fixed wavelength λ = 577.5 nm (*E* = 2.15 eV). (f) Intensity in the *xy* plane for a perturbed dense lattice at θ = 31.5°. Intensity in the *xz* plane for incidence angles: (g) θ = 30.5°, (h) θ = 31.5°, and (i) θ = 32.5°. (j) Field intensity enhancement maximum (|**E**_max_|^2^/|**E**_0_|^2^ as a function of θ for hexagonal dense lattice, rectangular empty superlattice, and dense perturbed lattice in orange, blue, and green, respectively. The specific chosen angles are marked and overplotted on the dispersion diagram in panel (a).

To understand the origin of the observed narrow dark mode at the Δ-point, we examine near-field distributions obtained from full-wave simulations (COMSOL). We employ a similar setup to the experiment, driving the structure with plane waves incident from the glass side. Importantly, as a starting point, we require a simulation of a fluorescence band structure near the Δ-point to pinpoint which exact (*k*_*y*_, ω) plane waves to use in COMSOL simulations to match the Δ-points precisely. For that, we use the *treams* reciprocity code, which is benchmarked against COMSOL to be accurate to within subpercent levels for angle- and frequency-resolved PLE maps, providing an 850-fold computing speed improvement. The result is shown in Figure 5a, where we clearly see the dark spot at Δ, as in the experiment (Figure 4c). We pick three radiation angles (in glass, *n* = 1.465), θ = (30.5, 31.5, 32.5)° that tune through the dark Δ-point, at which we perform the COMSOL simulations of near field distributions (at a wavelength of 577.5 nm (∼2.15 eV)). At an incidence angle of θ = 31.5°, the in-plane wavevector (*k*_∥_) closely matches the Δ-point (2π/3*a*) at a wavelength of 577.5 nm. In Figure 5b,c, for the dense hexagonal unit cell, the field is primarily concentrated near the particles, and there is no coupling into the waveguide mode. This is consistent with the fact that a subdiffractive lattice provides no wave vector matching. In stark contrast, the empty rectangular supercell (Figure 5d,e) at this angle shows a field intensity distribution (|**E**|^2^/|**E**_0_|^2^, normalized to **E**_0_ the field strength of the incident wave) which is typical for diffractive grating coupling from the far field into the waveguide.^61,62^ Notably, Figure 5f–i for the dense perturbed lattice shows a delicate cancellation mechanism for waveguide coupling only at the Δ-point. The reciprocal lattice of this perturbed structure equals that of the empty rectangular superlattice, meaning that waveguide coupling is wave vector allowed. However, right at the Δ-point, coupling to the waveguide mode nearly disappears. As shown in panel (h), although the field within the waveguide is significantly diminished at this angle, it is prominently enhanced right at the central particle in the unit cell. We interpret this formation of a high-*Q* mode with a strongly enhanced near field in the spirit of reported mechanisms behind the formation of bound states in the continuum. The dominant radiative loss for waveguide SLRs occurs within the waveguide mode. For the dense lattice at zero perturbation strength, this loss channel is strictly forbidden at all wave vectors, which can be viewed in real space terms as a destructive interference between the radiation from the center particle in the unit cell and that from the corner particles spanning the rectangular supercell. Upon perturbation, this destructive interference becomes imperfect and persists most prominently at the Δ-point. It is important to note that this does not represent a strict (quasi)-BIC mechanism, as out-of-plane radiation loss is not canceled. Additionally, as the angle is scanned through the Δ-point, the near field in the vertical cross cuts changes, forming a distinct two-lobe pattern in the phosphor layer at a 32.5° incidence (panel h). This shows that both TE and TM waveguide modes are engaged, with mode coupling occurring due to the plasmon particles.

To highlight the different field localizations, we compare the maximum localized field intensity enhancement |**E**_**max**_|^2^/|**E**_**0**_|^2^ in the *xy* plane, attained just outside the central particle, at different input angles of incidence θ while scanning through the Δ-point as shown in Figure 5j. The empty rectangular superlattice (blue curve) shows a dip in the maximum local field enhancement at the Δ-point, indicating that the field delocalization over the waveguide reduces the near-field intensity. In contrast, the dense hexagonal lattice (orange curve) maintains a nearly constant maximum field enhancement across all angles, suggesting that the field is more uniformly distributed regardless of the incidence angle. This behavior aligns with the absence of a waveguide coupling condition. The dense perturbed lattice achieves a maximum field enhancement of nearly 250, approximately four times higher than in the other cases, commensurate with the higher *Q*. This distinct behavior of the perturbed case highlights the influence of symmetry and mode interactions on field enhancement and coupling efficiency. This observation is consistent with theoretical works by refs (48, 56 and 71), which predict that bipartite plasmon lattices can be engineered to achieve significantly enhanced near fields and high-*Q* modes.

## Plasmon Lasing and Spontaneous Symmetry Breaking

**Figure 6:**
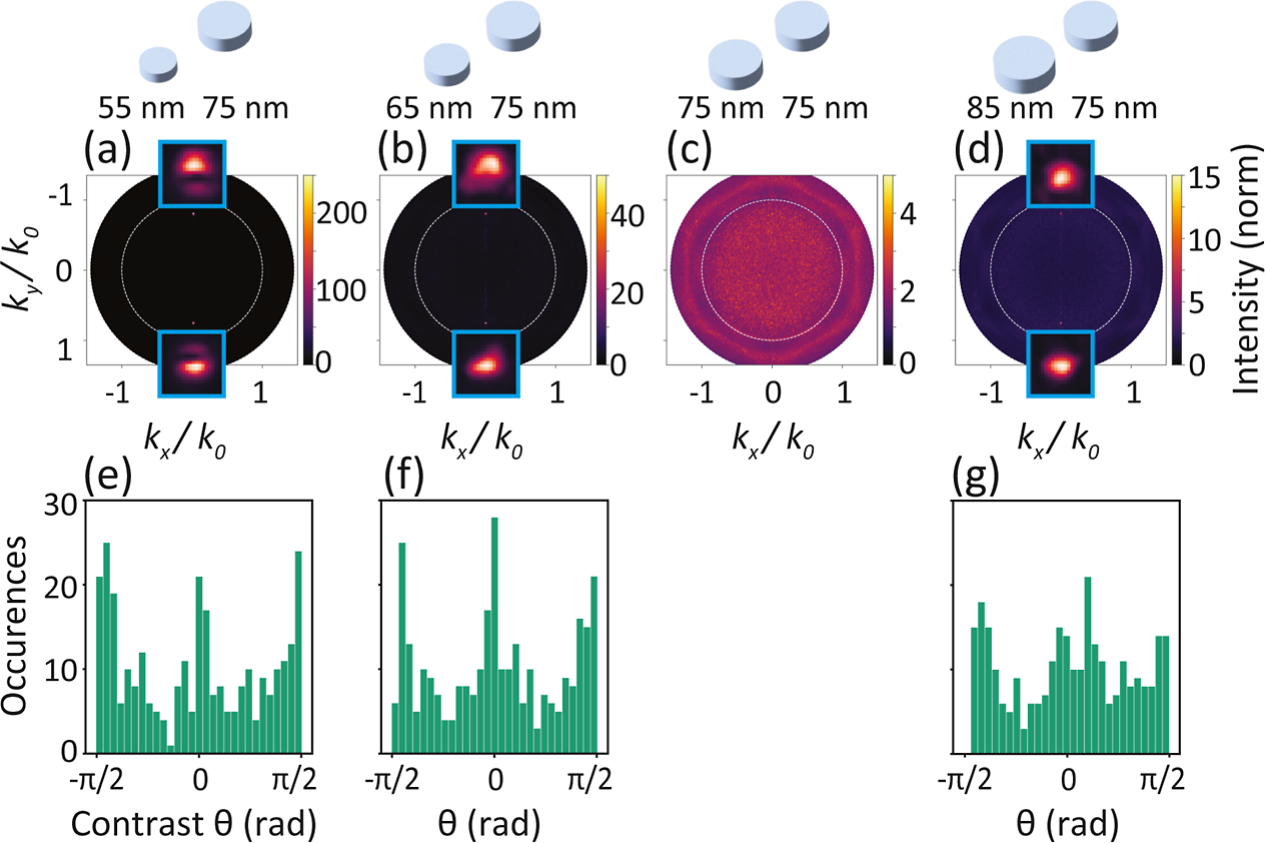
Fourier-space output above lasing threshold from bandfolded *K*-points. Panels (a,b,d) when the dense lattice is diameter-perturbed with a rectangular superlattice, lasing spots appear at the two Δ-points. Panel (c) above threshold, the unperturbed dense lattice displays random speckle patterns as opposed to uncorrelated Poisson statistics typical of fluorescence—a telltale sign of lasing. (e,f,g) the perturbed cases show random fluctuations due to spontaneous symmetry breaking in relative intensity between Δ and Δ′. (e–g) show histograms for time traces of 300 single-shot lasing measurements of the parameter θ, that maps the relative intensity of the two competing modes. Occurrences bunch toward pure Δ and Δ′ lasing (at θ = π/2 and −π/2), and to Δ + Δ′ lasing at θ = 0. The lasing data is normalized by fluorescent signal from a bare waveguide slab (as in a PL enhancement experiment).

Finally, we show that it is possible to study plasmon lasing in dense lattices, appearing as laser emission at the band folded *K*-point guided modes. In our setup, we have access to femtosecond pulsed pumping (515 nm) and can capture Fourier space emission maps in a single-shot fashion with a camera synchronized to the pump laser repetition rate (20 Hz).^18^ At low fluences, we observe fluorescence in Fourier space, as shown in the previous figures. However, when crossing a pump threshold fluence, we observe lasing in spectrally resolved input–output curves, and the Fourier images start to acquire hallmark features of spatial coherence. Examples of these Fourier images are shown in Figure 6 for the same perturbation series as in Figure 3. Input–output curves of the lasing for the various perturbation cases are shown in the Supporting Information. In the unperturbed case, Figure 6c, we observe that the below-threshold diffuse fluorescence image which has spatially uncorrelated shot noise is replaced by a high contrast, spatially correlated, speckle pattern,^72^ which evidence the emergence of coherence. On basis of the emission wavelength (measured as 579 nm) we attribute it to lasing at the *K*-points. Since the *K*-points are beyond the light cone, they are not directly visible. However, laser light is scattered out by residual disorder, e.g. from small particle size fluctuations, filling the back focal plane with speckle. In contrast, for nonzero perturbation (Figure 6a,b,d), the *K*-point lasing emission is folded into the light cone, and lasing spots appear exactly at the two Δ-points, i.e., at the folded *K*-points. Insets in Figure 6a,b,d show enlargements of the lasing spots. Particularly the 55–75 nm dense perturbed lattice (Figure 6a) displays the typical donut beam observed in many plasmonic and dielectric DFB laser works,^18,73,74^ pointing at the quasi-BIC nature of the lasing condition. Polarization topology of the vortex beam could reveal the quasi-BIC nature of the lasing mode involved, and therefore in future experiments we aim to measure polarization in Fourier space using a single-shot Stokes polarimetric setup. We have also observed lasing from dense perturbed lattice where the *M*-point is folded toward the Γ-point, with lattice constant *a* = 210 nm (see Supporting Information). To our knowledge, the dense plasmon lattices investigated in our work are the densest plasmon lattices so far reported to show lasing: standard Γ-point lasers with rhodamine-doped polymer waveguide layer have pitch of around 390 nm,^14^ and recently^75^ reported high-index InP layers with Au lattice with pitch of 270 nm.

Recently, we reported that diffractive plasmon lattice lasers of hexagonal symmetry present spontaneous symmetry breaking.^18^ In hexagonal lattices, the *K* and *K*′ points feature Bloch modes that are, by construction, degenerate in eigenfrequency and have identical mode patterns up to time-reversal/phase conjugation. Upon crossing the lasing threshold, we found spontaneous symmetry breaking both in relative amplitude between *K* and *K*′ lasing [parity symmetry] and in relative phase [*U*(1) symmetry] for such diffractive *K*-point lasers.^18^ This also occurs in dense lattices. In the single-shot lasing experiments with the dense perturbed lattices, large variations in intensity between the Δ and Δ′-points are apparent from shot to shot. This is in line with the notion of spontaneous symmetry breaking between the guided *K* and *K*′-points, since the three *K*-points that together constitute one of the lasing modes fold to the Δ-point, while the three *K*′-points that constitute the other lasing mode together fold onto the Δ′-point (Figure 1d). In the absence of the folding perturbation, the single-shot lasing data shows speckle patterns that vary from shot to shot, indicating that spontaneous symmetry breaking occurs also for beyond-light line operation.

We map the single-shot spontaneous symmetry breaking in relative intensity between Δ and Δ′ in the same phase space as reported in ref (18). To this end, from the summed pixel intensities of the two lasing spots, *I*_Δ_ and *I*_Δ′_, we calculate the parity symmetry breaking parameter θ as , which maps contrast between *K*/*K*′ lasing in terms of the polar angle of a unit sphere. The resulting histograms of occurrences of θ are presented in Figure 5e,f,g, and can be read as follows: at θ = 0 the Δ and Δ′-point are equally bright, whereas at θ = ± π/2 lasing is purely into Δ respectively Δ′-points. In all three perturbation cases, the spontaneous symmetry breaking leads to continuous distributions in the phase space, as in the diffractive lattice studied in ref (18). Lasing and spontaneous symmetry breaking for the folding strategy of Figure 2d (which preserves the dense lattice symmetry and repeats 6 *K*-points inside the light line) is reported in the Supporting Information. These observations show that band folding is an effective strategy to observe salient features of beyond-light line Bloch modes.

## Conclusion

In this work we have shown experimentally and by T-matrix calculations that the dispersion relation of guided modes of dense plasmon lattices can be accessed by bandfolding induced by introducing a supercell periodicity by slight modulations in particle diameter. We measure *Q*-factors of over 300 at the folded *K*-point of the dense perturbed lattice, three times higher than that of the rectangular empty superlattice. This observation highlights that radiation cancellation mechanisms that have been well studied in the context of quasi-BIC formation by symmetry breaking^11^ also arise in dense multipartite plasmon lattices.^76^ We can observe lasing from both the purely guided *K*-points in a dense plasmon lattice, and from band folded *K*-points. Yet, our work also highlights subtleties in the bandfolding strategy. First, the chosen supercell must be matched to the effective mode index of interest to ensure that the special points of interest actually fold into the light cone. Second, the strength of the perturbation is a trade off between making the features of interest sufficiently strongly visible (favoring raising the perturbation to raise the outcoupling strength) and avoiding changing the underlying band structure. Third, we observe that interpretation of the folded band structures is hard for two reasons. On the one hand, the band structures of the empty supercell is qualitatively similar to the folded bands of the dense lattices, with the dense nature of the folded lattices mainly expressing as larger mode splittings. On the other hand, the folded band structure is observed on top of the original scattering response that occurs within the light cone, giving rise to Fano resonances in observables (Figure 1d). This work offers opportunities to experimentally study dense plasmon lattices with gain.

## Methods

### Coupled Dipole Model

We employ the Ewald lattice summation technique for point dipole lattices as reported in ref (57, 58 and 77). We calculate the in-plane band structure (only *E* fields in *x* and *y* directions) by taking scatterers with negligible out-of-plane polarizability. For the polarizability we assume silver ellipsoids with aspect ratio (*x*, *y*, *z*) = (2.431, 2.431, 1), and use experimental permittivity data of silver presented in ref (78).

### Optical Setup

We use the frequency-doubled output of a Light Conversion Pharos laser as pump, with 515 nm wavelength and 250 fs pulse duration. Through an epi-lens and microscope objective (Nikon CFI Plan Apochromat lambda 100×, NA = 1.45) we illuminate the sample with a 70 μm diameter collinear beam. Pump input power is set by rotating halve-wave plate placed in front of a linear polarizer, and we reject it from the emission signal through a combination of 532 nm dichroic mirror and a 550 nm long pass filter. To create a Fourier image on our detection camera (Thorlabs CS2100M-USB), an insertable Fourier lens can be placed in focus of the back-focal-plane of the objective, via a 1:1: telescope. For single-shot lasing measurements, the electronic signal of the lasers’s pulse picker drives the camera. For fluorescence (below threshold) band structure measurements, we operate at a repetition rate of 1 MHz (multishot mode) but at low pump pulse power.

### Near Field Calculations

We use the finite element method (FEM) as implemented in COMSOL Multiphysics 5.2.0 to perform a three-dimensional simulation of the fabricated structure in response to far-field plane wave excitation. The computational domain spans the unit cell in the periodicity plane and extends a few wavelengths into both the substrate and superstrate. We apply Bloch-Floquet boundary conditions at the edges of the unit cell and periodic port conditions at the top and bottom along the illumination direction. Additionally, we employ perfectly matched layers (PMLs) on the exterior boundaries of the ports to absorb any unwanted reflections. The particles are meshed with a nominal element size of 5 nm, while other layers are meshed at 1/sixth of their wavelength, and the PMLs are meshed using a sweep distribution method.

## Supporting Information Available

Sample preparation (Section S1, SEM images Figure S1); Enhancement in 0 and 90° polarization (Section S2, Figure S2); Lorentzian fits for *Q* calculation (Section S3, Figure S3); Band structure simulations using *treams* (Section S4, Figure S4); Lasing input output curves and additional lasing data for (folded) dense perturbed lattices (Sections S5and S6, Figures S6–S8) (PDF)
