# Supplementary material for: Accessing Beyond-Light Line Dispersion and High-Q Resonances of Dense Plasmon Lattices by Bandfolding
Source: ACS Photonics. 2025 Jan 7;12(2):1163–73. doi: 10.1021/acsphotonics.4c02323 (PMC11843721; doi:10.1021/acsphotonics.4c02323)
Supplement: Supplementary file 1 — ph4c02323_si_001.pdf [file ph4c02323_si_001.pdf]

Supporting information for: Accessing beyond-light line dispersion and high-Q resonances of dense plasmon lattices by bandfolding

Nelson de Gaay Fortman,[†,¶] Debapriya Pal,[‡,¶] Peter Schall,[†] and A. Femius Koenderink*[,‡,§]

†*Institute of Physics, University of Amsterdam, 1098 XH Amsterdam, The Netherlands*

‡*Department of Physics of Information in Matter and Center for Nanophotonics, NWO-I Institute AMOLF, Science Park 104, NL1098XG Amsterdam, The Netherlands*

¶*Contributed equally*

§*Corresponding author*

E-mail: f.koenderink@amolf.nl

This supplement contains 15 pages and 8 figures.

1 Sample preparation

For electron beam lithography and lift off we spin 160 nm PMMA 495-A8 on base-piranha cleaned glass, deposit a 20 nm layer of Germanium (Ge) through thermal evaporation, and then spin a 60 nm CSAR AR-P 6200:09 layer that acts as high-resolution e-beam resist. We expose the CSAR layer using a Raith Voyager 50 KeV at a nominal dose of 140 $\mu C/cm^2$. Once the CSAR resist layer is developed, we etch the Ge and PMMA layers using SF_6 and O_2 plasma after developing the resist layer. This provides for high-resolution holes in the Ge, yet a wide opening in the PMMA that is conducive to liftoff. We then evaporate 35 nm silver (Ag) at a deposition rate of 0.1 nm/s and perform a liftoff process in warm acetone. Scanning electron microscope (SEM) images of the samples are shown in Fig S1. Finally, we apply a roughly 0.45 μm thick SU8-based polymer film, doped with 0.5 wt% rhodamine 6G, which functions as the fluorescent waveguide layer.

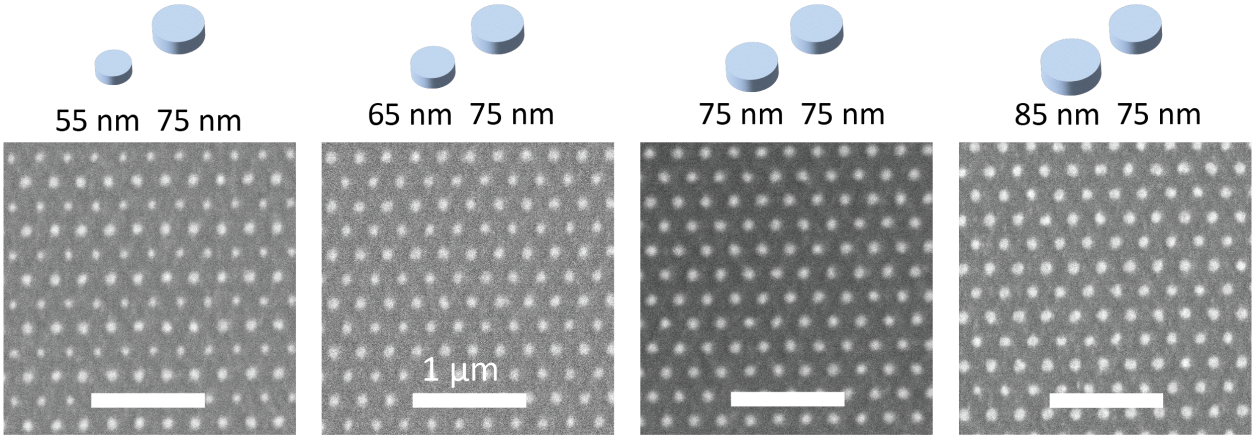

Figure S1: **SEM images.** Scanning electron micrographs of unperturbed (c) and perturbed (a,b,d) dense hexagonal lattices.

2 Enhancement in 0 and 90 degree polarization

Additional to the fluorescence enhancement band structure measurements in the main manuscript (Fig 3), we record perturbation sweeps in s and p scattering planes by placing a polarizer in front of the spectrometer slit. The results are shown in Fig S2, showing that different bands

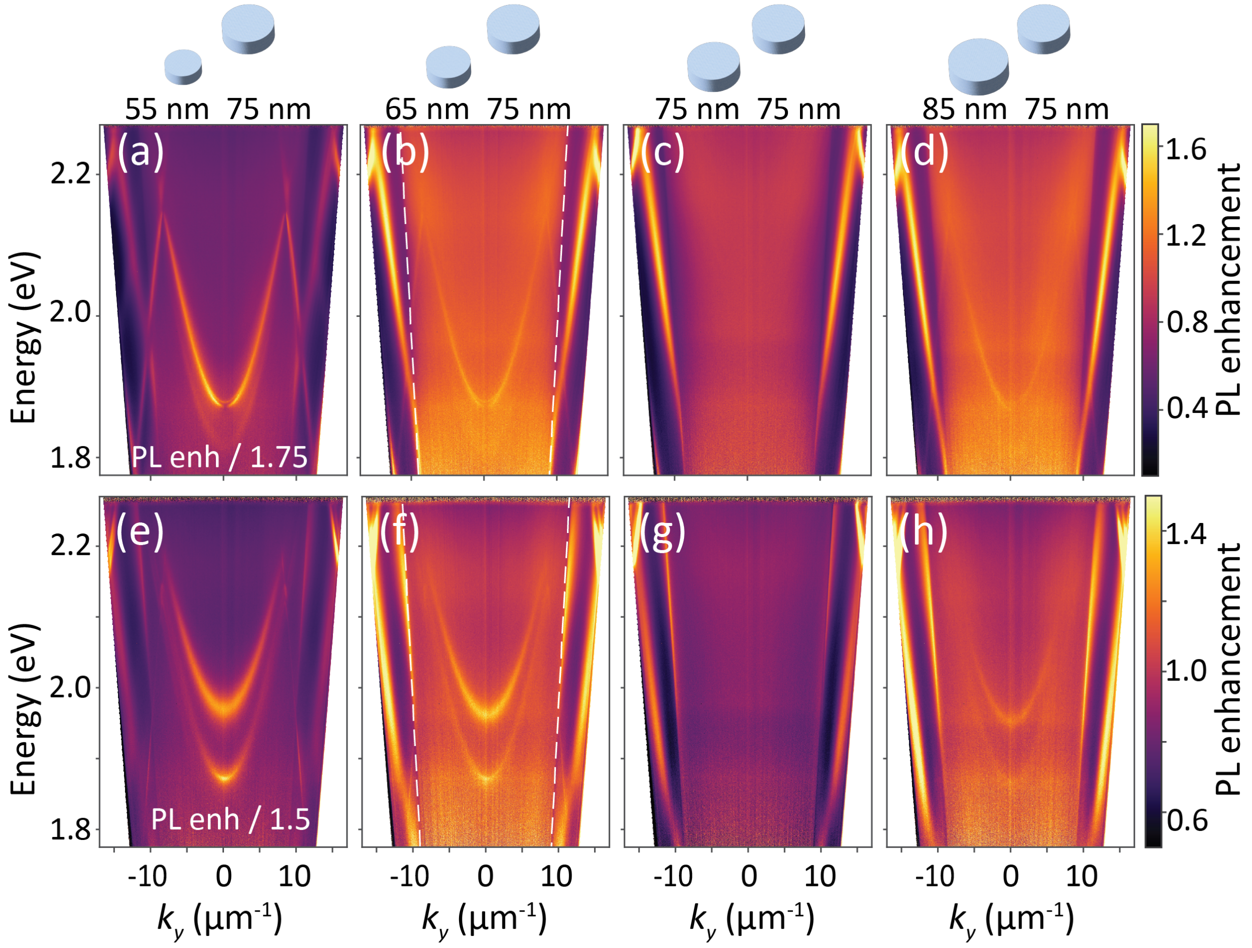

Figure S2: **Polarization dependent measurements.** Fluorescence enhancement band structure measurements with linear polarizer placed in front of spectrometer slit. Panels (a-d): polarizer oriented horizontally, and panels (e-h): vertically.

appear based on polarizer orientation. The TE/TM mode assignment in the main text is based on the assignment rules explained in,[1,2] which are accurate near the $k_{||} = 0$ line.

3 Lorentzian fits for Q calculation

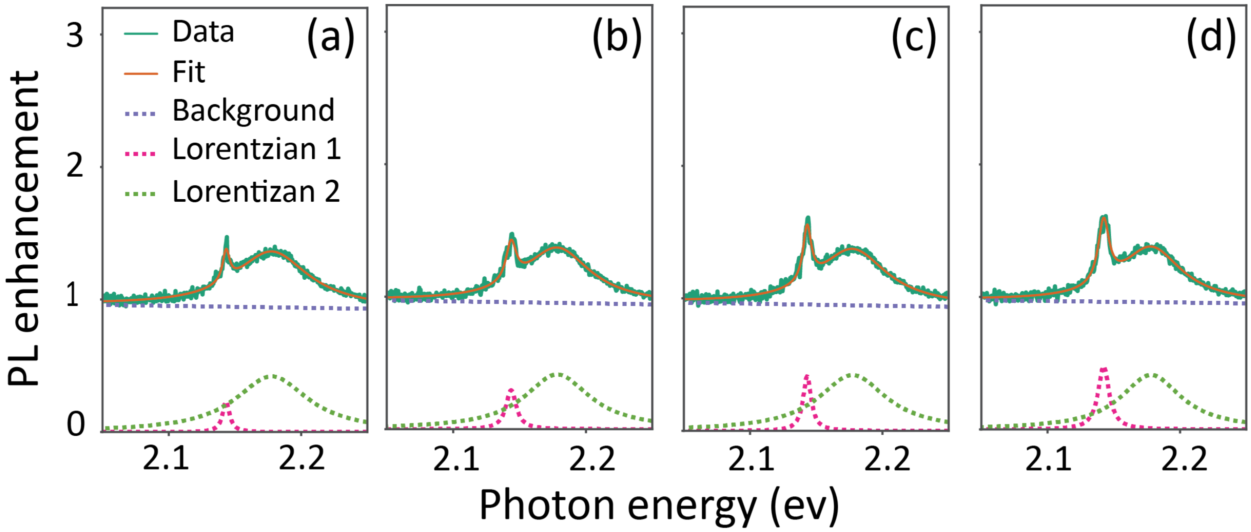

Figure S3: **Lorentzian peak fitting.** Four vertical crosscuts through the fluorescence enhancement band structure measurement (main Fig 4(b,c)), from $k_y = 8.55\ \mu\text{m}^{-1}$ (a) until $k_y = 8.60\ \mu\text{m}^{-1}$ (d), each with two fitted Lorentzian peaks and linear background. (a) Q = 334, (b) Q = 228, (c) Q = 285, (d) Q = 212.

The measured fluorescence enhancement spectra display narrow bands near the high symmetry points (main Fig 4(c)). To calculate the quality factors, we take crosscuts along the energy axis near these dispersions and fit curves that are parametrized as the sum of two Lorentzian peaks, plus allowing for a linearly sloping background. The fit proceeds using the Python package lmfit (Levenberg-Marquardt algorithm). The model fits the Lorentz center frequencies, widths, and peak amplitudes. We start the fit procedure for crosscuts well away from the high symmetry point, and then fit crosscuts increasingly close to the symmetry point using the fitted parameters from the previous cross cut as starting guesses for the next. Example fits that allow to judge the quality of fit are shown in Figure S3. The smallest fitted spectral width corresponds to Q=334 in Fig S3(a), where $\lambda = 578.6$ nm and

spectral width $d\lambda = 1.73$ nm. This width is still a factor 4 higher than our instrument's spectral resolution resolution (Andor Shamrock 163i, with 300 lines per mm grating and 25 μm wide slit).

4 Band structure simulations using *treams*

We utilize full-wave simulations to predict fluorescence band diagrams of dense plasmonic lattice structures without any of the following approximations: nearly-free photon dispersion, absence of multipolar interactions, absence of substrates, and perturbative nature of the supercell. We use the open-source software code *treams* based on the T-matrix method by Beutel, Frenandez-Corbaton and Rockstuhl.[3] To calculate the emission properties, we use reciprocity to relate plane wave incident calculations of absorption to far-field angular emission. According to the Helmholtz reciprocity principle, light absorption at a given position originating from the far field is reciprocally linked to the emission of light from the same position toward the far field at the same angle and polarization.[4–7] By employing this method, we can estimate angularly resolved far-field emission and the radiative part of the system's local density of optical states (LDOS). The *treams*-method combines the single object T-matrix with Ewald lattice summation to solve for the two-dimensional periodic arrangement of particles and then employs a multilayer S-matrix formalism to consider stratification, thereby accounting for the layered dielectric system in which the particles are embedded. To set up *treams* we first use the finite element solver JCMsuite to calculate the T-matrices of individual scatterers within a homogeneous medium of index equal to that of the polymer. We assume a 450 nm thick polymer with a refractive index of $n = 1.6$ on glass (n=1.465) and air (n=1) above. To implement the reciprocity method, we assume a numerically very small tracer loss (k = 0.006) in the polymer, excluding the 35 nm thick layer containing the particles, which must remain lossless for technical reasons. We calculate the volume-integrated absorption enhancement in the polymer layer as a proxy for

photoluminescence enhancement (PLE). To map out the dispersion diagrams, we use two linear polarizations (s- and p-polarization) to illuminate from the glass side at various polar angles with $k_x = 0$ for different wavelengths. We plot the polarization averaged absorption, normalized to a reference calculation without particles, as a metric for photoluminescence enhancement (PLE). Benchmarking with FEM simulations with periodic boundary conditions demonstrates better than percent level agreement while achieving an 850-fold speed up.

To obtain a library of T-matrices as function of wavelength and size for nanocylinders we use JCMsuite, which has built-in features to evaluate the multipole decomposition of the scattered field. The refractive index of the cylinder is modeled using Drude's model $\varepsilon(\omega) = \varepsilon_\infty - \omega_p^2/\omega(\omega + i\gamma)$ for silver (Ag) with $\epsilon_\infty = 5.43 + 0.55i$, $\omega_p = 1.39 \cdot 10^{16}$ rad/s, and $\gamma = 8.21 \cdot 10^{13}$ rad/s.[8] Since the T-matrix calculation involves expanding plane waves into vector spherical wave functions, the surrounding environment of the cylinder is considered lossless. We consider multipole order of cylinders up to ($l_{\max} = 5$), i.e., up to the 32 pole or triacontadipolar response. For the lattice sum, we consider propagating diffraction orders ($G_{\max}$) up to a maximum radius of 5 times the reciprocal lattice vector in the reciprocal space.

First, we present the calculated dispersion diagrams for two reference particle array configurations, as illustrated in Figure S4. The first configuration is the unperturbed sub-diffractive hexagonal lattice, referred to as the "dense lattice" from here on, shown in panels (a) and (b). The second configuration is the rectangular diffractive reference lattice, named the "empty superlattice" in panels (c) and (d). Following this, we discuss the dispersions that involve band folding, specifically in "dense perturbed lattices," which are shown in panels (e), (f), (g), and (h). The first Brillouin Zone of the dense hexagonal lattice ($a = 250$ nm) in Figure S4a, has the K, K' points at its corners just outside the light cone of the glass (outer dotted circle), rendering them inaccessible and truly guided. Thus, there are also no dispersive bands within the air light line in the predicted PL enhancement (Figure S4b).

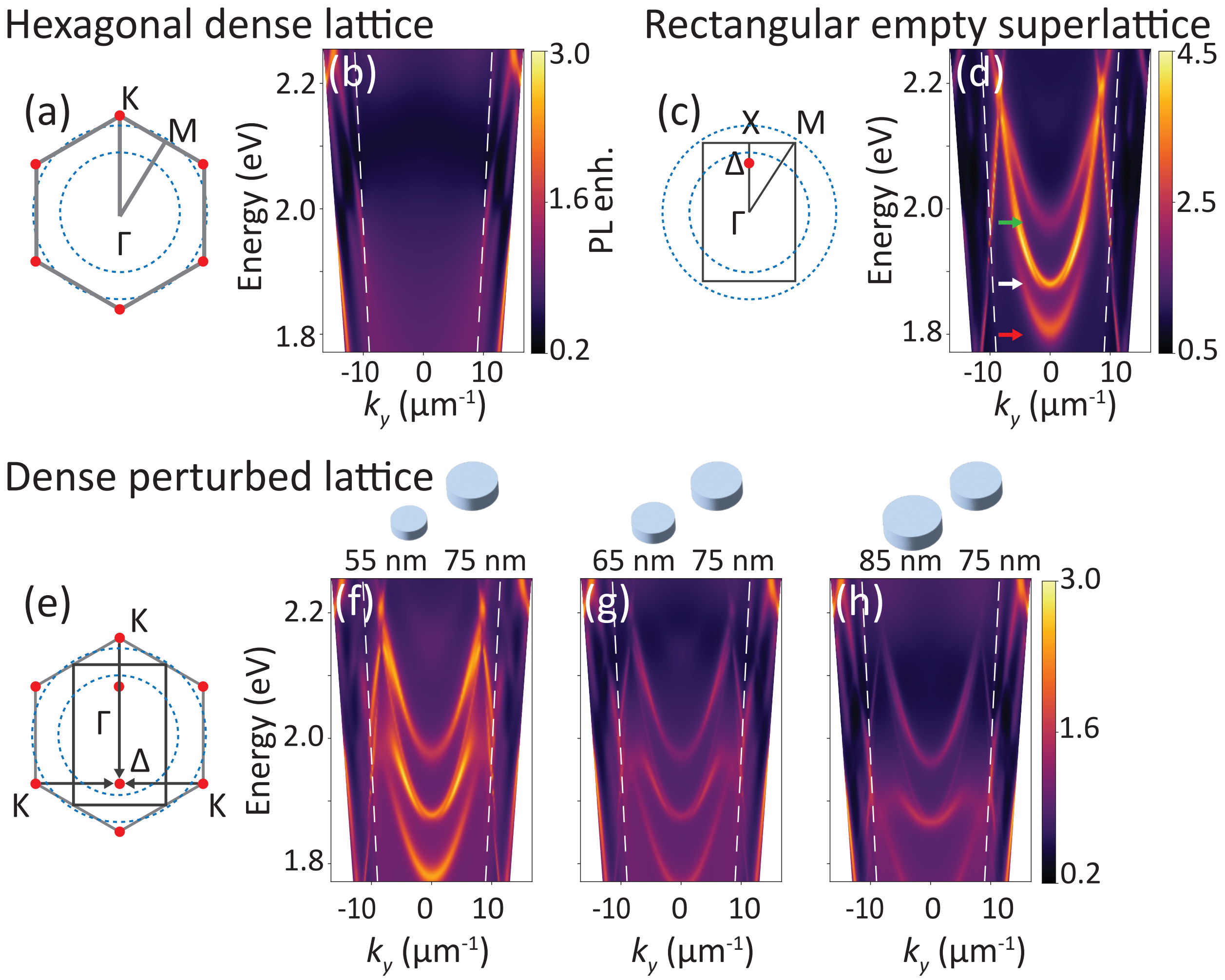

Figure S4: ***treams*-simulated band structures.** Reciprocal lattice and simulated dispersion band diagrams using reciprocity for (a-b) a hexagonal dense lattice and (c-d) a rectangular empty lattice, both with a lattice constant of 250 nm and 75 nm diameter nanocylinders. Green arrow: TM waveguide modes. White and red arrow: TE bands. In the bottom panel (e-h), the dense perturbed lattice features two particles per unit cell: one with a fixed diameter of 75 nm and the other with varying diameters of (f) 55 nm, (g) 65 nm, and (h) 85 nm (as shown in the insets).

A weak but broad dark band, with slight dispersion near 2.1 eV, indicates the absorptive plasmon resonance. Somewhat surprisingly, a set of bands emerges at intermediate wave vectors (effective index $n = 1.2$, i.e., quasiguide modes). They can be understood using an effective medium picture: similar modes arise in systems where a thin metal partial reflector is placed between the glass and the waveguiding polymer.

Figure S4(c) shows the 1st BZ for the rectangular empty superlattice, which is itself rectangular and entirely situated within the light line of the glass substrate (indicated by the dotted outer circle). At this large periodicity, the folded waveguide mode dispersion that falls within the frequency range of interest appears as parabolic bands centered at the Γ points, as shown in Figure S4(d). Also shown are band crossings at the so-called Δ-points, which are situated between the Γ- and X-points. The underlying guided modes are those of the 2D polymer layer, which supports a single transverse electric (TE) and transverse magnetic (TM) polarized mode, with an average mode index of around 1.54.[9] Colored arrows indicate the contribution of both fundamental TM (green arrow, around 2 eV) and TE modes. The TM waveguide mode has a weak in-plane electric field and is not strongly scattered by the anisotropic, oblate aspect ratio cylindrical plasmonic particles, which primarily have in-plane polarizability. This results in the TM feature being only weakly visible. In contrast, the TE mode is dominantly polarized in the plane of the particles. Consequently, the TE band experiences mode splitting, as indicated by the white and red arrows.

Having established the main features expected for the dense lattice and for the empty superlattice, we now examine the folded band structure of the dense perturbed lattice: the hexagonal system where we introduce a perturbation in the particle diameter for every other row. The results of weak superlattice perturbations with a diameter difference of only 10 nm are shown in Figure S4(f-g). The folded bands appear as expected and are only visible as faint features due to the weak perturbation. When the perturbation strength is increased to a 20 nm diameter difference, as illustrated in Figure S4(e), the folded bands become more prominent and exhibit higher contrast. Compared to the rectangular supercell, we observe

that the folding does not introduce entirely new bands *per se*; rather, the folded bands of the hexagonal dense lattice qualitatively resemble the bands of the rectangular empty superlattice, as seen in Figure S4(d). However, a closer inspection shows that the band splittings are significantly different and generally larger. This difference can be qualitatively attributed to the fact that the dense perturbed lattices contain a higher polarizability, which leads to increased scattering per unit cell. Finally, it is important to note that with larger diameter differences, the perturbation not only folds the bands but also changes the band splitting. This is particularly notable with perturbed particles that are enlarged to 85 nm, which display a much larger band splitting compared to cases with smaller perturbations.

5 Lasing for dense perturbed lattices

Input-output curves. In the main manuscript, we claim the results of Fig 6 are actual lasing output, and as evidence we display in SI Fig S5 input-output curves of the lasing wavelength (579 nm), as measured with the spectrometer (exposure time 300 ms, reprate is 20 Hz, so 15 single shots in one camera capture, with 2x2 software pixel binning).

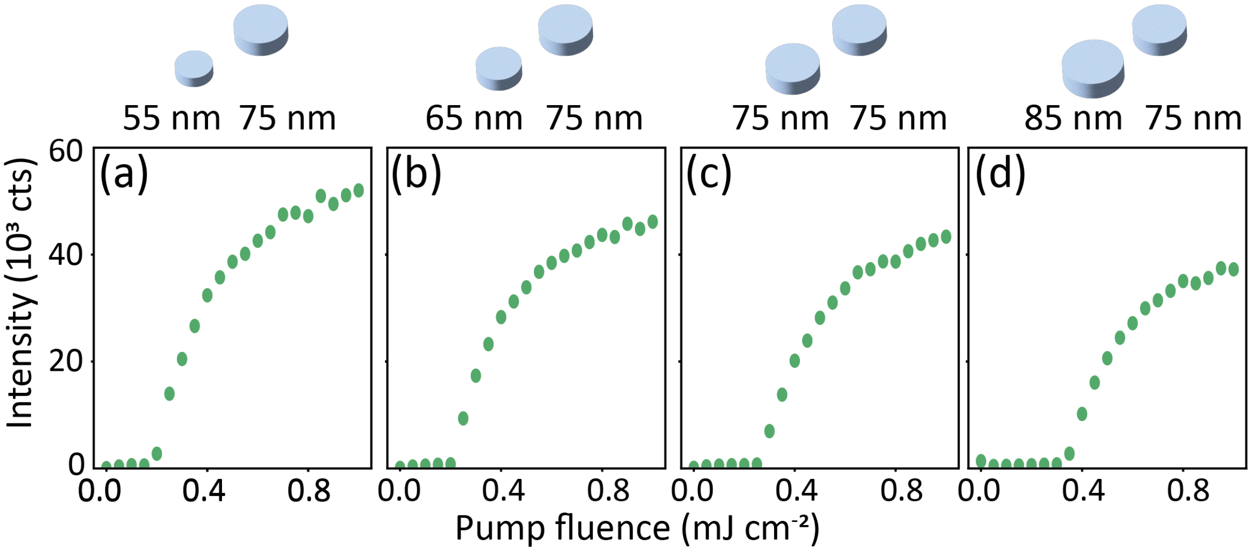

Figure S5: **Lasing input-output curves.** Input-output curves for the dense lattice (c), and the various dense perturbed lattices (a), (b), (d).

Densest plasmon lattice laser. We show lasing not only from band folded K-points in dense perturbed lattices (main Fig 6) of pitch 250 nm, but also for the same perturbation geometry but pitch 210 nm. In this configuration, two M-points fold toward the Γ-point. In terms of antenna areal density this is the densest plasmon lattice laser reported in literature, to the best of our knowledge.

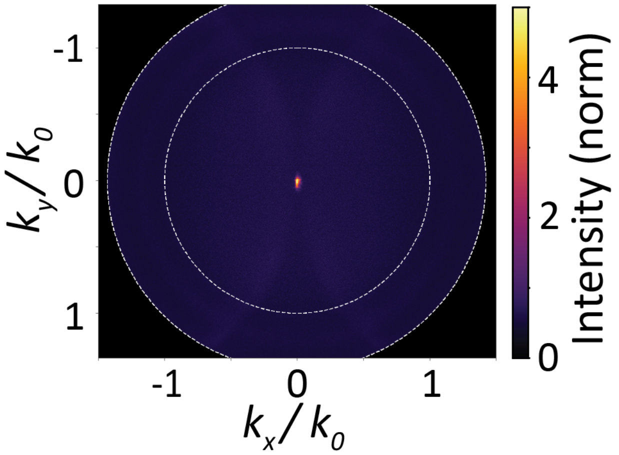

Figure S6: **Densest plasmon lattice laser.** Densest lattice laser at the Γ-point (folded from the M-point). The pitch is 210 nm.

6 Different folding strategy: folding from K to K

Fluorescence enhancement band structures. In the main manuscript Fig 3 and 4, we displayed measured results for band folding strategy where three K-points are folded to one Δ-point (and K' to Δ'), explained in detail in to the first row of main Fig 2. Here, in SI Fig 7, we show it is also possible to employ the second folding strategy, where K folds towards K of the supercell (panels (a,b)). The first row of experimental band structure data, panels (c-f), shows polarization averaged fluorescence enhancement. As expected, we observe no photonic Bloch modes for the dense lattice, panel (e) (same data as main Fig 3(c)). Also in this folding strategy, when we apply superlattice perturbations, the measured band structures contain signal of folded photonic bands, their contrast depending on perturbation strength. The contrast of the bands is less pronounced, which might be attributed to the fact that in this

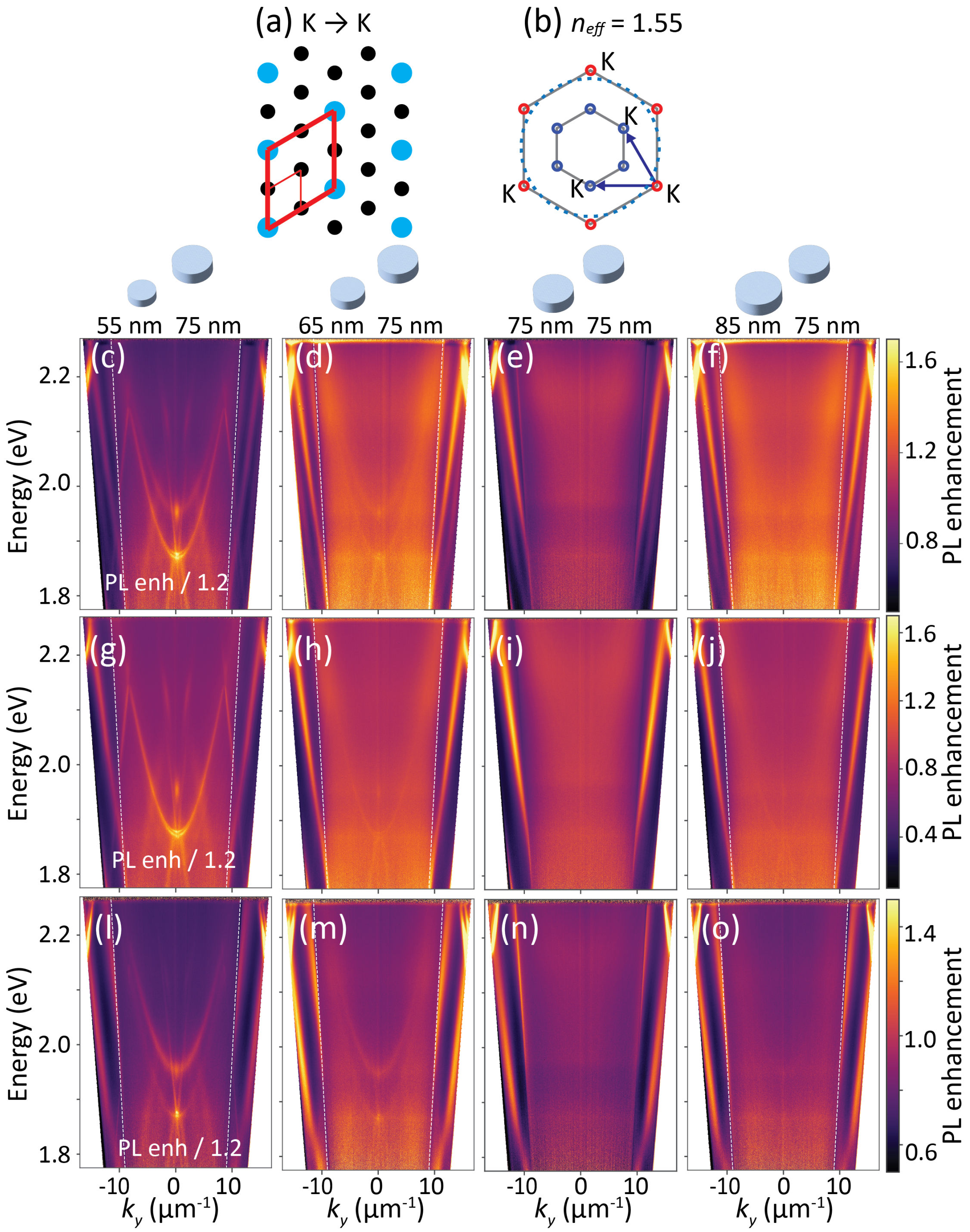

Figure S7: **Measurements of bandfolding from K to K.** (a) Folding strategy where K folds to K, obtained by applying diameter perturbations of double period along the symmetry axes of the hexagonal dense lattice. (b) Three K-points of the dense lattice fold toward three K-points of the dense perturbed lattice. K' folds towards K'. (c-f) Fluorescent enhancement band structures containing emission in all polarization states. (g-j) Band structures with horizontally oriented polarizer in front of spectrometer slit. (l-0) Band structures with vertically oriented polarizer.

case, half of the metal is perturbed, compared with K-Δ folding. In panel (c), dispersions near the Γ-point immediately draw attention, where large band splitting occurs both in the parabolic and linear bands. However, more importantly, inspecting panel (c) and (d), it is evident that the dispersions near the K-point are very alike those bandfolded dispersions near the Δ-point in main Fig 3. This is further highlighted by the results in the second and third rows of experimental data are measurements with a polarizer placed before the spectrometer slit, either horizontally oriented (panels (g-j)) or vertically oriented (panels (l-0)): again, the dispersions near K are very similar to bandfolded dispersions near Γ in SI Fig 2. This important observation highlights the fact that one can employ different folding strategies to uncover the same beyond-the-light line dispersions. Also in this dataset, a dark spot is observed at the bandfolded K-point, especially visible in panel (g).

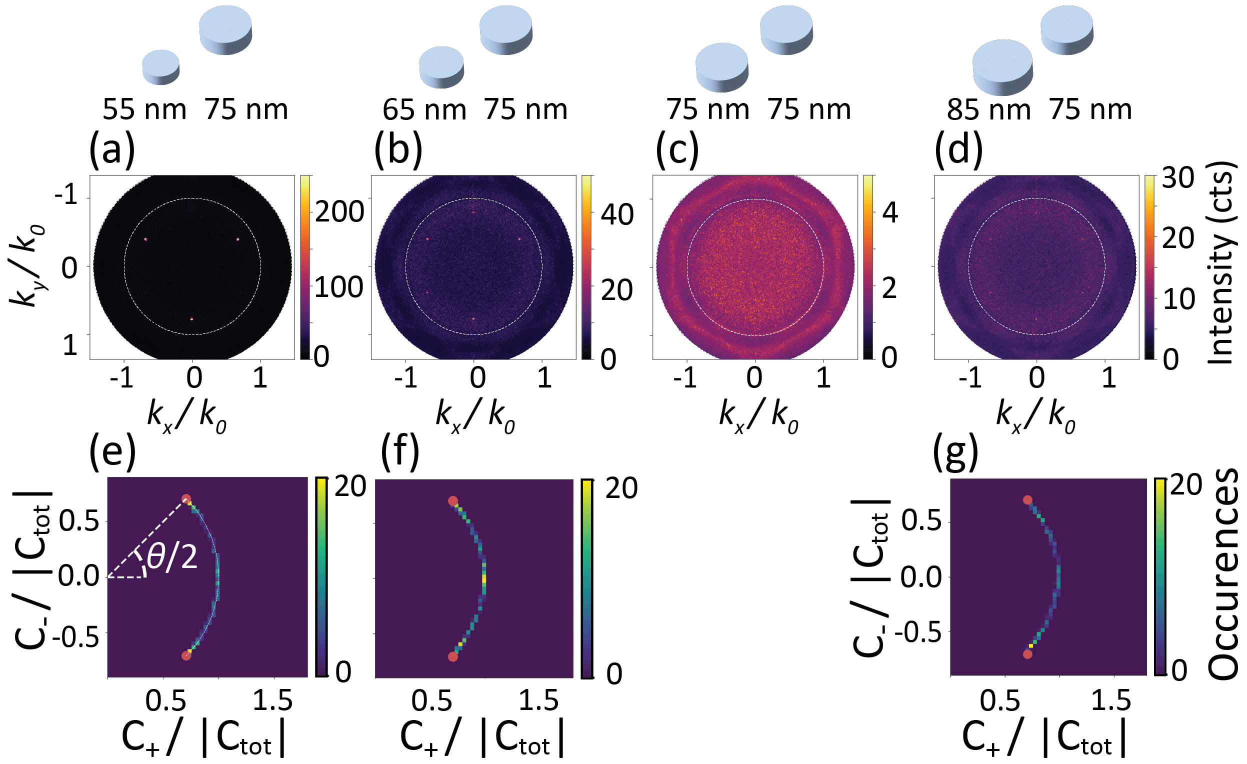

Figure S8: **Lasing and spontaneous symmetry breaking for bandfolding from K to K.** (a-d) lasing data for various superlattice diameter perturbations. (e-g) histograms of parity symmetry breaking, with parameter θ as measure for contrast of K/K' mode lasing. The lasing spot signals are fluorescence background subtracted, by subtracting a same-sized patch directly next to each spot, to generate the histogram plots.

Lasing and spontaneous symmetry breaking. We study lasing and spontaneous symmetry breaking (SSB) for the K to K bandfolding strategy. Starting with the dense lattice, SI Fig 8(c) (same data as main Fig 6(c)), the spatially coherent random speckle patterns find their origin in truly guided K-mode lasing. Upon applying superlattice diameter perturbations, the guided K-modes are bandfolded to the K dispersions of the dense perturbed lattices, which support lasing radiation to the far-field. For 10 nm perturbations, panels (b) and (d), the lasing signal is slightly brighter than that of the speckle pattern, but for the 55 nm - 75 nm perturbation case (panel (a)), very sharp lasing intensity peaks are observed.

For each sample we recorded 300 single-shots of lasing output, and we found similar behavior as we recently reported for a diffractive plasmon lattice K-point laser.[10] That is, from shot to shot, the relative intensity between the K and K' laser modes varies randomly, which we attribute to an incomplete bifurcation in a spontaneous symmetry breaking event. With the same methods as described in Ref.,[10] we project the intensity data to a subspace of lasing in superpositions of the K and K' modes. The six-vector of lasing intensities in the 6 output spots I (obtained by summing intensity in a small area around each $K^{(\prime)}$-point) is projected on vectors that span K/K', to produce coefficients: $C_+ = \langle I|v_+\rangle$ and $C_- = \langle I|v_-\rangle$ with $v_\pm = 1/\sqrt{6}(1, \pm 1, 1, \pm 1, 1, \pm 1)$. The resulting histograms of panels (e-g) show all datapoints lie on the arc between the two points $C_+ = \pm C_- = \sqrt{1/2}$ (indicated by red dots). The angle θ of a datapoint on the arc is the spontaneous parity symmetry breaking parameter, and maps to the surface of the unit sphere as polar coordinate. The fact that no datapoints occur within the arc means that the system does not lase in other spot combinations than those corresponding to superpositions of K/K' modes. What is more, in panel (e) as in main Fig 6(e-g), bunching towards the center and towards edges of the arc is observed, which indicates the system either favors K+K' lasing or pure K/K' lasing.

References

(1) Schokker, A. H.; van Riggelen, F.; Hadad, Y.; Alù, A.; Koenderink, A. F. Systematic Study of the Hybrid Plasmonic-Photonic Band Structure Underlying Lasing Action of Diffractive Plasmon Particle Lattices. *Phys. Rev. B* **2017**, *95*, 085409.

(2) Guo, K.; Koenderink, A. F. Spatial Intensity Distribution in Plasmonic Particle Array Lasers. *Phys. Rev. Appl.* **2019**, *11*, 024025.

(3) Beutel, D.; Fernandez-Corbaton, I.; Rockstuhl, C. *Treams* – a T-matrix-based Scattering Code for Nanophotonics. *Comput. Phys. Commun.* **2024**, *297*, 109076.

(4) Bharadwaj, P.; Deutsch, B.; Novotny, L. Optical Antennas. *Adv. Opt. Photon.* **2009**, *1*, 438–483.

(5) Carminati, R.; Sáenz, J. J.; Greffet, J.-J.; Nieto-Vesperinas, M. Reciprocity, Unitarity, and Time-Reversal Symmetry of the S Matrix of Fields Containing Evanescent Components. *Phys. Rev. A* **2000**, *62*, 012712.

(6) Janssen, O. T. A.; Wachters, A. J. H.; Urbach, H. P. Efficient Optimization Method for the Light Extraction from Periodically Modulated LEDs Using Reciprocity. *Opt. Express* **2010**, *18*, 24522–24535.

(7) Bailly, E.; Hugonin, J.-P.; Coudevylle, J.-R.; Dabard, C.; Ithurria, S.; Vest, B.; Greffet, J.-J. 2D Silver-Nanoplatelets Metasurface for Bright Directional Photoluminescence, Designed with the Local Kirchhoff's Law. *ACS Nano* **2024**, *18*, 4903–4910.

(8) Penninkhof, J. J.; Sweatlock, L. A.; Moroz, A.; Atwater, H. A.; van Blaaderen, A.; Polman, A. Optical Cavity Modes in Gold Shell Colloids. *J. Appl. Phys.* **2008**, *103*, 123105.

(9) Urbach, H. P.; Rikken, G. L. J. A. Spontaneous Emission from a Dielectric Slab. *Phys. Rev. A* **1998**, *57*, 3913–3930.

(10) de Gaay Fortman, N.; Kolkowski, R.; Pal, D.; Rodriguez, S. R. K.; Schall, P.; Koenderink, A. F. Spontaneous Symmetry Breaking in Plasmon Lattice Lasers. *Sci. Adv.* **2024**, *10*, eadn2723.
